# Perioperative management of adults with traumatic brain injury

**DOI:** 10.1177/17504589231187798

**Published:** 2023-08-31

**Authors:** Chinazo Okeke, Jenny Zhang, Tom Bashford, Matthew Seah

**Affiliations:** 1 Department of Surgery, University of Cambridge, Cambridge, UK; 2 Division of Anaesthesia, University of Cambridge, Cambridge, UK

**Keywords:** Traumatic brain injury, Cerebral oxygenation, Intracranial pressure, Cerebral perfusion pressure, Hypoxia, Hypothermia

## Abstract

Despite advances in management strategy, traumatic brain injury remains strongly associated with neurological impairment and mortality. Management of traumatic brain injury requires careful and targeted management of the physiological consequences which extend beyond the scope of the primary impact to the cranium. Here, we present a review of the principles of its acute management in adults. We outline the procedure which patients are assessed and the critical physiological variables which must be monitored to prevent further neurological damage. We describe current interventional strategies from the context of the underlying physiological mechanisms and recent clinical data and identify persisting challenges in traumatic brain injury management and potential avenues of future progress.

## Introduction

Traumatic brain injury (TBI) encompasses a diverse collection of intracranial injuries following impact to the cranium. This review aims to highlight the current perioperative guidelines in the United Kingdom with regard to the management of the adult patient with TBI and secondary prevention of further damage. TBI is the most prominent cause of morbidity and mortality in people aged one to 40 in the United Kingdom, and had an aggregate cost of £5.1 billion in the United Kingdom in 2010, with an average cost per case of £11,340 (Parsonage 2016). Damage sustained from TBI can be grouped into primary injuries, including haematomas and traumatic axonal injury (TAI) sustained due to the initial trauma and secondary injuries which develop some time after, ranging from minutes to days. TBI may be viewed as a progressive injury, with optimal patient recovery contingent upon reducing the magnitude of the secondary insult.

## Incidence

The Global Burden of Diseases, Injuries and Risk Factors (GBD) study has estimated the age-standardised worldwide incidence of TBI to be 369 per 100,000 people in 2016 (James et al 2019). However, the true consequences of TBI extend beyond its incidence and can also be considered from the perspective of disease burden, which can be assessed using the measures of years of lost life (YLLs) and years lived with disability (YLDs). The GBD study (James et al 2019) reported that TBI was the cause of an estimated 8.1 million YLLs in 2016. Consistent with its high global incidence, TBI remains one of the commonest causes of death and disability in the United Kingdom. Data from NICE (2014) have indicated that 1.4 million people annually from England and Wales attend emergency departments with acute head injury.

A crucial part of public health prevention strategies is to reduce the risk of incurring the initial trauma. Prevention strategies identified by the Lancet Neurology Commissions on TBI (Maas et al 2022) include attention to road traffic safety, fall prevention in the elderly and implementation of measures to mitigate risks in contact sports.

However, once the injury has occurred, the clinical focus is on maintaining metabolic normality for surviving cerebral tissue, while seeking to prevent further secondary injury. Here we present the principles of acute management of TBI and the perioperative considerations.

## Acute management

The acute management phase of TBI is crucial to stabilising the patient and preventing any secondary brain injury resulting from physiological insults such as ischaemia, reperfusion injury and hypoxia (Moppett 2007).

## Patient assessment

Initial patient assessment is carried out in accordance with Advanced Trauma Life Support (ATLS) guidelines. An important component of the assessment is the Glasgow Coma Score (GCS) to estimate the extent of injury and provide a rough approximation of morbidity and mortality risk of the patient (Moppett 2007). Full cervical spine immobilisation should be performed for patients with any of the following risk factors: GCS less than 15 on initial assessment, neck pain or tenderness, focal neurological deficits, paraesthesia in the extremities and/or any other clinical suspicion of cervical spine injury (NICE 2019). In patients with reduced consciousness (GCS equal to or less than eight), there should be early involvement of an anaesthetist or critical care physician to establish a secure airway (eg: endotracheal intubation) (NICE 2019).

Endotracheal intubation is considered the gold standard, but it is essential to assess the benefits, risks and timing of this on a case-by-case basis. For example, patients who are unable to maintain SpO_2_ > 90% with supplemental oxygen, who are agitated and who are suspected of having cerebral herniation where maintaining adequate oxygenation is a concern, should also be considered for intubation (Sönmez 2022). While anaesthetic agents allow for quick control of the airway and modulation of intracranial pressure (ICP) through CO_2_ control, many are also potent vasodilators resulting in an undesirable drop in mean arterial pressure (MAP) (Dinsmore 2013). To maintain cerebral perfusion pressure (CPP), care must be taken to maintain the MAP during induction of anaesthesia, largely through the use of accompanying vasoconstrictors, such as metaraminol. Therefore, the need to induce anaesthesia must be balanced against the risk of decreasing blood pressure.

Effective pain management is also essential as uncontrolled pain may lead to a rise in ICP. The patient should be provided with reassurance if conscious, any fractures splinted and pain managed with small doses of intravenous opioids titrated against clinical response and baseline cardiorespiratory measurements recorded (NICE 2019).

The time elapsed before intervention is a major determinant of outcome (Trivedi et al 2022). Major Trauma Networks operate to ensure that the three overlapping phases of the patient’s journey (pre-hospital, in-hospital and post-hospital) are well-organised, so that patients are treated at the time and place which has the greatest benefit for them (Moran et al 2018).

## Imaging

Computed tomography (CT) is the core imaging modality in acute TBI. Early imaging helps to detect serious life-threatening abnormalities, allowing for an appropriate treatment plan to be developed. The following guidelines are the criteria recommended by NICE for performing CT imaging in TBI (NICE 2019) (see Table 1).

**Table 1:** This table outlines the risk factors used to determine the time interval within which a CT scan should be performed.

For adults with the following risk factors, CT should be performed within one hour of risk factor being identified:	For adults with any of the following risk factors who have experienced some loss of consciousness or amnesia since the injury, a CT scan should be performed within eight hours of the TBI:
GCS less than 13 on initial assessment in the emergency department	Age 65 years or older
GCS less than 15 at two hours after the injury on assessment in the emergency department	Any history of bleeding or clotting disorders
Suspected open or depressed skull fracture	Dangerous mechanism of injury (a pedestrian or cyclist struck by a motor vehicle, an occupant ejected from a motor vehicle or a fall from a height of greater than 1m or five stairs)
Any sign of basal skull fracture (haemotympanum, ‘panda’ eyes, cerebrospinal fluid leakage from the ear or nose, Battle’s sign)	More than 30 minutes’ retrograde amnesia of events immediately before the head injury
Post-traumatic seizure	
Focal neurological deficit	
More than one episode of vomiting	

CT: computed tomography; TBI: traumatic brain injury; GCS: Glasgow Coma Score.

## Management of raised ICP

**Figure 1:**
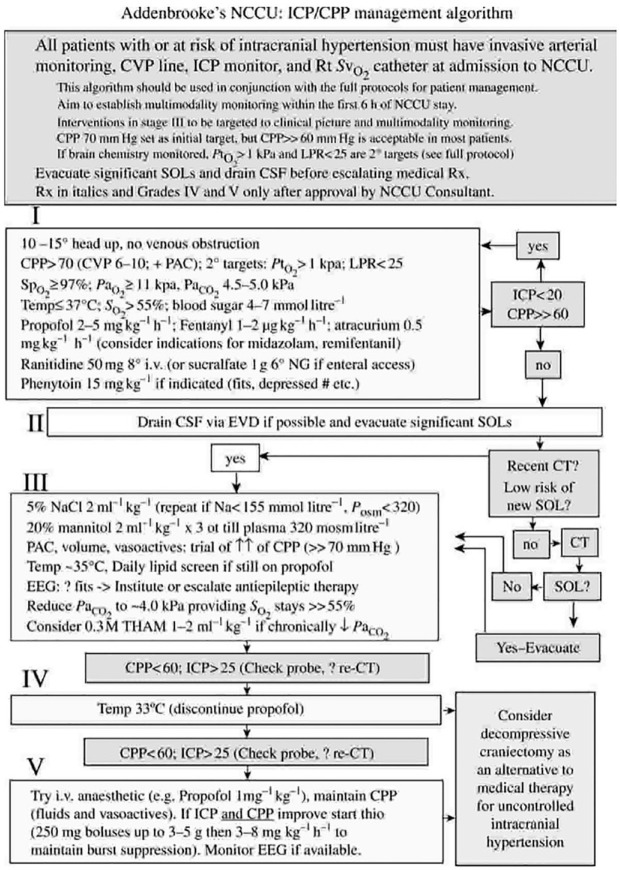
An example of an ICP/CPP management algorithm used by Addenbrooke’s Hospital, Cambridge

As the brain is contained within a non-expandable case of bone, the Monro-Kellie doctrine describes the sum of the volumes of the cerebrospinal fluid (CSF), intracranial blood and CSF must be constant, such that an increase in one necessitates a decrease in either or both of the other two (Mokri 2001). An increase in ICP can therefore impair cerebral blood flow and cause secondary ischaemia (Haider et al 2018) (see Figure 1).

ICP monitoring remains the mainstay of cerebral monitoring in these patients. A range of interventions exist to manage elevated ICP, with evidence that standardised protocols improve management and outcomes.

## Head elevation

Raising the patient’s head reduces ICP by displacing CSF from the intracranial department and promoting venous outflow. The head of the bed should be raised to 30°, with the patient’s head in a midline position to avoid compression of the internal jugular vein (Schizodimos et al 2020). Cerebral blood flow is unaffected (Feldman et al 1992). Head elevation above 45° should be avoided to prevent paradoxical increases in ICP in response to a fall in cranial perfusion pressure (CPP) (Moraine et al 2000). Care is taken in the polytrauma patient, particularly with spinal fracture(s), to protect other organ systems. In this instance, a reverse Trendelenberg position can be used to maintain spinal alignment, while still allowing the head to be raised.

## Hyperventilation

Hyperventilation refers to an elevated minute alveolar ventilation, which decreases the intraarterial carbon dioxide partial pressure (PaCO_2_), resulting in cerebral vasoconstriction and a consequent decrease in ICP (Grubb et al 1974). PaCO_2_ levels which rise above normal (5.2kPa) result in cerebral vasodilatation and an increase in ICP. PaCO_2_ values which are uncontrolled may increase the risk of cerebral haemorrhage (Deng et al 2020).

However, hyperventilation is also associated with numerous adverse effects. If PaCO_2_ levels fall below 4.2kPa, cerebral vasoconstriction can start to limit cerebral perfusion, while hyperventilation has also been associated with electrolyte imbalances, such as hypokalaemia and hypocalcaemia (Godoy et al 2017). Furthermore, hyperventilation may cause respiratory alkalosis, increasing the oxygen affinity of haemoglobin and reducing oxygen delivery to peripheral tissues (Curley et al 2010). These explain why this intervention is not recommended prophylactically and is typically reserved for brief periods of acute neurological dysfunction in severe TBI.

## Hyperosmolar therapy

Intermittent boluses of a hyperosmolar agent such as mannitol lower ICP via two distinct mechanisms (Dinsmore 2013, Peters et al 2018). First, the formation of an osmotic gradient across the blood-brain barrier causes water to move from the parenchyma into the intravascular space. Second, mannitol reduces blood viscosity, thereby promoting plasma expansion. Due to autoregulation, plasma vasoconstriction occurs, decreasing cerebral blood volume.

However, mannitol is also associated with a range of adverse effects, such as arterial hypotension and rebound of cerebral oedema (Shi et al 2020). Therefore, hypertonic saline is increasingly being used instead (Gu et al 2018).

## Hypothermia

Inducing hypothermia reduces cerebral metabolic demands. Selective brain cooling can be achieved by using a cooling cap, while systemic cooling can be achieved with a cooling blanket or endovascular catheters. Treatment outcomes may be related to the time taken to reach the goal temperature, when the cooling began and the duration of cooling (Finkelstein & Alam 2010). Some studies suggest that longer periods of cooling may be associated with more favourable outcomes, as cerebral swelling and oedema are greatest three to five days after injury (Fox et al 2010). However, the adverse effects associated with hypothermia, such as coagulation disturbances, may counteract its positive effects, and thus there is lack of support for the use of therapeutic hypothermia as a first-tier treatment (Sandestig et al 2014).

## Glycaemic control

Hyperglycaemia is associated with poor outcomes following TBI (Jeremitsky et al 2005). Hyperglycaemia may arise as a consequence of the catecholamine surge and may be further exacerbated by the administration of exogenous steroids and catecholamines. Monitoring is required to ensure blood sugar levels are maintained between 4mmol/L and 8mmol/L (Dash & Chavali 2018).

The importance of blood glucose monitoring was illiustrated in a seminal single-centre study by Van de Berghe (2001) which showed that intensive glycaemic control reduced mortality in the surgical intensive care unit (ICU) setting. However, subsequent randomised controlled trials (RCTs) identified that hypoglycaemia was also associated with a substantial increase in mortality (Godoy et al 2017). Therefore, patients with TBI are particularly sensitive to both hyperglycaemia as well as hypoglycaemia.

## Neurosurgical interventions

Surgical intervention may be required to evacuate an epidural or subdural hematoma. The local mass effect of hematomas may cause an increase in ICP and clinical signs such as mental status changes, dilated pupils or extensor posturing consistent with brainstem herniation. Subdural hematomas which are over 10mm in size or causing more than 5mm midline shift may be considered for operative intervention (Fomchenko et al 2018).

A craniectomy is a further surgical procedure which can be used to reduce ICP. This involves the removal of part of the skull, which allows the brain to swell without increasing the ICP. Options for the repair of the cranial defect include the use of the previously removed bone flap or the use of a variety of synthetic implants. While there is still no consensus on the timing of the repair, some institutions report performing this after at least three months to allow the swelling to subside (Martin et al 2014).

## Fluid intervention and blood pressure management

Secondary brain injury may arise from episodes of hypotension after TBI and are associated with worse patient outcomes. To maintain cerebral perfusion and intravascular volume, intravenous fluids are commonly administered, with the choice traditionally being 0.9% saline, as it is relatively isotonic to plasma. Isotonic solutions do not increase brain water content as they do not change plasma osmolality and distribute freely in the extracellular fluid compartment (Alvis-Miranda et al 2014). However, a systematic review and meta-analysis found that 0.9% saline and low-molecular weight hydroxyethyl starch were associated with lower mortality than other intravenous solutions, such as balanced crystalloid solutions (Tseng et al 2020). In addition, hypertonic crystalloids may be commonly used in patients with cerebral edema and raised ICP.

Vasopressor therapy is also often administered in combination with intravenous fluid administration. Although noradrenaline is most commonly used, there has been concern that noradrenaline infusion may result in hypoperfusion to other organs as a consequence of excessive vasoconstriction. A systematic review was unable to show a significant benefit associated with noradrenaline infusion in patients with TBI (Lloyd-Donald 2020).

## Seizure management

Seizures typically happen where there is tissue damage that arises as a consequence of the injury (Englander et al 2014). The tissue damage results in scarring, which alters astrocyte physiology and can cause neurotransmitter imbalance (Robel 2016). Seizure risk is reduced by the use of anti-epileptic medications in the first seven days (Wiles 2022). Although phenytoin is commonly used, it is associated with several side effects which include liver enzyme induction, cardiac arrhythmias and hypotension. Due to its low incidence of adverse effects, levetiracetam has become increasingly popular, with a meta-analysis demonstrating that levetiracetam showed similar efficacy to phenytoin in patients with TBI (Fang et al 2021).

## Hypoxia

With cessation of blood flow, the intracellular production of adenosine triphosphate is reduced. The subsequent reduction in primary active transporter ion channels results in the intracellular accumulation of sodium ions and cytotoxic edema. Furthermore, ischaemia also promotes the release of glutamate, which activates *N*-methyl-d-aspartate (NMDA) channels and promotes calcium influx. Calcium activates lytic enzymes and promotes free radical formation and cell death.

In contrast to the well-known association between hypoxia and adverse outcomes in TBI, the influence of hyperoxia is less certain. A systematic review demonstrated that hyperoxia was not associated with an increase in mortality (Ni et al 2019). It is reasonable to tolerate a degree of hyperoxia after TBI to allow adequate oxygen reserves for unanticipated events, especially in light of the established dangers of hypoxia to the injured brain.

## Recent progress and challenges in TBI management

In 2017, the Lancet Neurology published its first commission on TBI (Maas et al 2022). This drew attention to the largely unrecognised public health challenge caused by TBI and called for a concerted approach to better understand its origins and mechanisms. Since then, there have been several large-scale observational studies, such as CENTRE-TBI and TRACK-TBI, which highlighted prognostic factors such as frailty, comorbidities and alcohol misuse (Lecky et al 2021, Steyerberg et al 2019).

There is the need to develop new prediction tools to stratify TBI patients for more personalised care (Chinnery 2022). A potential new approach could be to measure novel CSF and serum biomarkers which could, in the future, become easy-to-use biochemical tests. While there has clearly been progress over the past five years, many advances are yet to achieve routine clinical implementation. Avenues of future research could involve identifying subgroups of patients which would be most likely to benefit from certain interventions and developing greater understanding of the effects of genetic variation on the biology of TBI to facilitate individualised management.

## Conclusion

The management of TBI poses many challenges, as TBI does not a comprise a single disease entity, but rather a collection of discrete disease types. Despite a diverse arsenal of management strategies, TBI continues to be associated with neurological impairment and mortality, with an estimated half of the global population experiencing at least one TBI during their lifetime (Young & Hughes 2020). The wide range of treatment strategies further emphasises the complexity of treating TBI, especially as the specific context of each injury and patient variability must be taken into account. Despite improving and evolving management strategies, the global burden of TBI is increasing, so health care systems must continue to adapt (Feigin et al 2019).

## References

[bibr1-17504589231187798] Alvis-Miranda HR Castellar-Leones SM Moscote-Salazar LR 2014 Intravenous fluid therapy in traumatic brain injury and decompressive craniectomy Bulletin of Emergency and Trauma 2 (1) 3–14 Available at https://www.ncbi.nlm.nih.gov/pmc/articles/PMC4771253/27162857 PMC4771253

[bibr2-17504589231187798] Chinnery PF 2022 Traumatic brain injury advances since 2017: What has changed? The Lancet Neurology 21 (11) 953–954 36183710 10.1016/S1474-4422(22)00337-4

[bibr3-17504589231187798] Curley G Kavanagh BP Laffey JG 2010 Hypocapnia and the injured brain: More harm than benefit Critical Care Medicine 38 (5) 1348–1359 20228681 10.1097/CCM.0b013e3181d8cf2b

[bibr4-17504589231187798] Dash HH Chavali S 2018 Management of traumatic brain injury patients Korean Journal of Anesthesiology 71 (1) 12–21 29441170 10.4097/kjae.2018.71.1.12 PMC5809702

[bibr5-17504589231187798] Deng R-M Liu Y-C Li J-Q Xu J-G Chen G 2020 The role of carbon dioxide in acute brain injury Medical Gas Research 10 (2) 81–84 32541133 10.4103/2045-9912.285561 PMC7885708

[bibr6-17504589231187798] Dinsmore J 2013 Traumatic brain injury: An evidence-based review of management Continuing Education in Anaesthesia Critical Care & Pain 13 (6) 189–195

[bibr7-17504589231187798] Englander J Cifu DX Diaz-Arrastia R 2014 Seizures and traumatic brain injury Archives of Physical Medicine and Rehabilitation 95 (6) 1223–1224 24862307 10.1016/j.apmr.2013.06.002 PMC4516165

[bibr8-17504589231187798] Fang T Valdes E Frontera JA 2021 Levetiracetam for seizure prophylaxis in neurocritical care: A systematic review and meta-analysis Neurocritical Care 36 (1) 248–258 34286461 10.1007/s12028-021-01296-z

[bibr9-17504589231187798] Feigin VL Nichols E Alam T , et al 2019 Global, regional, and national burden of neurological disorders, 1990–2016: A systematic analysis for the Global Burden of Disease Study 2016 The Lancet Neurology 18 (5) 459–480 30879893 10.1016/S1474-4422(18)30499-X PMC6459001

[bibr10-17504589231187798] Feldman Z Kanter MJ Robertson CS , et al 1992 Effect of head elevation on intracranial pressure, cerebral perfusion pressure, and cerebral blood flow in head-injured patients Journal of Neurosurgery 76 (2) 207–211 1730949 10.3171/jns.1992.76.2.0207

[bibr11-17504589231187798] Finkelstein RA Alam HB 2010 Induced hypothermia for trauma: Current research and practice Journal of Intensive Care Medicine 25 (4) 205–226 20444735 10.1177/0885066610366919

[bibr12-17504589231187798] Fomchenko EI Gilmore EJ Matouk CC Gerrard JL Sheth KN 2018 Management of subdural hematomas: Part II. Surgical management of subdural hematomas Current Treatment Options in Neurology 20 (8) 34 30019165 10.1007/s11940-018-0518-1

[bibr13-17504589231187798] Fox JL Vu EN Doyle-Waters M Brubacher JR Abu-Laban R Hu Z 2010 Prophylactic hypothermia for traumatic brain injury: A quantitative systematic review CJEM 12 (4) 355–364 20650030 10.1017/s1481803500012471

[bibr14-17504589231187798] Godoy DA Seifi A Garza D Lubillo-Montenegro S Murillo-Cabezas F 2017 Hyperventilation therapy for control of posttraumatic intracranial hypertension Frontiers in Neurology 8 250 28769857 10.3389/fneur.2017.00250 PMC5511895

[bibr15-17504589231187798] Grubb RL Raichle ME Eichling JO Ter-Pogossian MM 1974 The effects of changes in PaCO2 on cerebral blood volume, blood flow, and vascular mean transit time Stroke 5 (5) 630–639 4472361 10.1161/01.str.5.5.630

[bibr16-17504589231187798] Gu J Huang H Huang Y Sun H Xu H 2018 Hypertonic saline or mannitol for treating elevated intracranial pressure in traumatic brain injury: A meta-analysis of randomized controlled trials Neurosurgical Review 42 (2) 499–509 29905883 10.1007/s10143-018-0991-8

[bibr17-17504589231187798] Haider MN Leddy JJ Hinds AL Aronoff N Rein D Poulsen D Willer BS 2018 Intracranial pressure changes after mild traumatic brain injury: A systematic review Brain Injury 32 (7) 809–815 29701515 10.1080/02699052.2018.1469045 PMC6192525

[bibr18-17504589231187798] James SL Theadom A Ellenbogen RG , et al 2019 Global, regional, and national burden of traumatic brain injury and spinal cord injury, 1990–2016: A systematic analysis for the Global Burden of Disease Study 2016 The Lancet Neurology 18 (1) 56–87 30497965 10.1016/S1474-4422(18)30415-0 PMC6291456

[bibr19-17504589231187798] Jeremitsky E Omert LA Dunham CM Wilberger J Rodriguez A 2005 The impact of hyperglycemia on patients with severe brain injury The Journal of Trauma: Injury, Infection, and Critical Care 58 (1) 47–50 10.1097/01.ta.0000135158.42242.b1 15674149

[bibr20-17504589231187798] Lecky FE Otesile O Marincowitz C , et al 2021 The burden of traumatic brain injury from low-energy falls among patients from 18 countries in the CENTER-TBI Registry: A comparative cohort study PLOS Medicine 18 (9) e1003761 34520460 10.1371/journal.pmed.1003761 PMC8509890

[bibr21-17504589231187798] Lloyd-Donald P 2020 In adult patients with severe traumatic brain injury, does the use of norepinephrine for augmenting cerebral perfusion pressure improve neurological outcome? A systematic review Injury 51 (10) 2129–2134 32739152 10.1016/j.injury.2020.07.054

[bibr22-17504589231187798] Maas AIR Menon DK Manley GT , et al 2022 Traumatic brain injury: Progress and challenges in prevention, clinical care, and research The Lancet Neurology 21 1004–1060 36183712 10.1016/S1474-4422(22)00309-X PMC10427240

[bibr23-17504589231187798] Martin KD Franz B Kirsch M Polanski W Von der Hagen M Schackert G Sobottka SB 2014 Autologous bone flap cranioplasty following decompressive craniectomy is combined with a high complication rate in pediatric traumatic brain injury patients Acta Neurochirurgica 156 (4) 813–824 24532225 10.1007/s00701-014-2021-0

[bibr24-17504589231187798] Mokri B 2001 The Monro-Kellie hypothesis: Applications in CSF volume depletion Neurology 56 (12) 1746–1748 11425944 10.1212/wnl.56.12.1746

[bibr25-17504589231187798] Moppett IK 2007 Traumatic brain injury: Assessment, resuscitation and early management British Journal of Anaesthesia 99 (1) 18–31 17545555 10.1093/bja/aem128

[bibr26-17504589231187798] Moraine J-J Berré J Mélot C 2000 Is cerebral perfusion pressure a major determinant of cerebral blood flow during head elevation in comatose patients with severe intracranial lesions? Journal of Neurosurgery 92 (4) 606–614 10761649 10.3171/jns.2000.92.4.0606

[bibr27-17504589231187798] Moran CG Lecky F Bouamra O , et al 2018 Changing the system – major trauma patients and their outcomes in the NHS (England) 2008–17 Eclinicalmedicine 2–3 (2) 13–21 10.1016/j.eclinm.2018.07.001 PMC6537569 31193723

[bibr28-17504589231187798] NICE 2014 Introduction. In: Head injury: Assessment and early management Guidance NICE Available at https://www.nice.org.uk/guidance/cg176/chapter/Introduction

[bibr29-17504589231187798] NICE 2019 Key priorities for implementation. In: Head injury: Assessment and early management Guidance NICE Available at https://www.nice.org.uk/guidance/cg176/chapter/Key-priorities-for-implementation#criteria-for-performing-a-ct-head-scan

[bibr30-17504589231187798] Ni Y-N Wang Y-M Liang B-M Liang Z-A 2019 The effect of hyperoxia on mortality in critically ill patients: A systematic review and meta analysis BMC Pulmonary Medicine 19 (1) 53 30808337 10.1186/s12890-019-0810-1 PMC6390560

[bibr31-17504589231187798] Parsonage M 2016 REPORT: An economic analysis traumatic brain injury and offending Available at https://www.centreformentalhealth.org.uk/sites/default/files/2018-09/Traumatic_brain_injury_and_offending.pdf

[bibr32-17504589231187798] Robel S 2016 Astroglial scarring and seizures The Neuroscientist 23 (2) 152–168 27118807 10.1177/1073858416645498

[bibr33-17504589231187798] Sandestig A Romner B Grände P-O 2014 Therapeutic hypothermia in children and adults with severe traumatic brain injury Therapeutic Hypothermia and Temperature Management 4 (1) 10–20 24660099 10.1089/ther.2013.0024 PMC3949439

[bibr34-17504589231187798] Schizodimos T Soulountsi V Iasonidou C Kapravelos N 2020 An overview of management of intracranial hypertension in the intensive care unit Journal of Anesthesia 34 (5) 741–757 32440802 10.1007/s00540-020-02795-7 PMC7241587

[bibr35-17504589231187798] Shi J Tan L Ye J Hu L 2020 Hypertonic saline and mannitol in patients with traumatic brain injury Medicine 99 (35) e21655 32871879 10.1097/MD.0000000000021655 PMC7458171

[bibr36-17504589231187798] Sönmez BM 2022 Chapter 5 – Management of traumatic brain injury from the aspect of emergency department and case studies. In: Rajendram R Preedy VR Martin CR (Ed) Diagnosis and Treatment of Traumatic Brain Injury Amsterdam, Elsevier Available at https://www.sciencedirect.com/science/article/pii/B9780128233474000348#bb0030 (Accessed September 2022)

[bibr37-17504589231187798] Steyerberg EW Wiegers E Sewalt C , et al 2019 Case-mix, care pathways, and outcomes in patients with traumatic brain injury in CENTER-TBI: A European prospective, multicentre, longitudinal, cohort study The Lancet Neurology 18 (10) 923–934 31526754 10.1016/S1474-4422(19)30232-7

[bibr38-17504589231187798] Peters NA Farrell LB Smith JP 2018 Hyperosmolar therapy for the treatment of cerebral edema. US Pharm 43 (1) 8–11. Available at https://www.uspharmacist.com/article/hyperosmolar-therapy-for-the-treatment-of-cerebral-edema#:~:text=Hyperosmolar%20therapy%20is%20a%20mainstay

[bibr39-17504589231187798] Trivedi DJ Bass GA Forssten MP , et al 2022 The significance of direct transportation to a trauma center on survival for severe traumatic brain injury European Journal of Trauma and Emergency Surgery 48 2803–2811 35226114 10.1007/s00068-022-01885-3 PMC9360055

[bibr40-17504589231187798] Tseng C-H Chen T-T Wu M-Y Chan M-C Shih M-C Tu Y-K 2020 Resuscitation fluid types in sepsis, surgical, and trauma patients: A systematic review and sequential network meta-analyses Critical Care 24 (1) 693 33317590 10.1186/s13054-020-03419-y PMC7734863

[bibr41-17504589231187798] Van den Berghe G Wouters P Weekers F , et al 2001 Intensive insulin therapy in critically ill patients New England Journal of Medicine 345 (19) 1359–1367 11794168 10.1056/NEJMoa011300

[bibr42-17504589231187798] Wiles MD 2022 Management of traumatic brain injury: A narrative review of current evidence Anaesthesia 77 (S1) 102–112 35001375 10.1111/anae.15608

[bibr43-17504589231187798] Young JT Hughes N 2020 Traumatic brain injury and homelessness: From prevalence to prevention The Lancet Public Health 5 (1) e4–e5 31806488 10.1016/S2468-2667(19)30225-7

